# McComber’s Goods

**Published:** 1874-12

**Authors:** 


McComber's Goods.—A few certificates, among a large number given without solicitation to this gentleman, may interest the reader. That they state the honest conviction of the writers we are well aware, and we believe every person certifying will cheerfully take the trouble to answer any inquiries upon the subject which may reach him by mail :

"The boots and shoes for our household, ladies included, made on the McComber principle, and at his establishment, have given such entire satisfaction as to comfortableness of fit, from the first hour of wearing them, and they are so substantially made, to say nothing of the neatness of their fitting, that it is a pleasure to commend them to the patronage of the public of both sexes, and of every age and condition in life."—Editor of Hall's Journal of Health.

From E. H. Gibbs, M.D., Publisher of Hall's Journal of Health :

"Joel McComber's patent boots and shoes must commend themselves to every anatomist who will carefully study their construction with reference to the human foot, and the duties which are imposed upon it. He is clearly very far in advance of all others in this important specialty. His boots are entirely comfortable from the first hour, and are honestly made of only choice materials. They are at once the most symmetrical, the most comfortable, and the cheapest foot-clothing to be found."

From Hon. Frank Fuller, formerly Governor of Utah :

"I have been seeking for perfect boots for a quarter of a century, and have patronized all the so-called 'improvements' to be found. Each one has been more incorrect in principle, and more uncomfortable in practical use, than the other ; but I find in McComber's system what seems to me to be the true principle. I do not see how the tenderest foot can be distorted by a boot made upon his last, or would I believe serious discomfort to be possible under his method. For myself, I shall continue to clothe my feet with his $16 boots just as long as I am able to pay for them. I would say the same if his price was $100 a pair. I say this most cheerfully, and I have not less than three reasons for saying it—first, because they are the handsomest boots I ever wore; second, because they are the most comfortable; and, third, because one pair of them will outwear two or three pairs of any other make."—Frank Fuller, 39 West Twenty-sixth Street, New York.

From L. Baily, the well-known leather dealer, Spruce Street, New York.

If the pedestrian, Weston, had worn a pair of shoes made on McComber's patent last, he could have walked the five hundred miles on time, and without chafing his feet. I have had my boots and shoes made for many years by a boot and shoemaker whose name has been before the New York public for fifty years, and at the time of his death he was the oldest shoemaker in the city; but with all his experience, never made me a pair of boots or shoes in which I walked as comfortably (even after the breaking in as it is commonly called among shoemakers) as I have walked from the first hour I put a boot or shoe upon my feet made upon the McComber last. I have a good platform or sole to stand upon, which remains under the bearings of the foot, and the upper-leather presses the foot evenly on every part without straining the leather, as well as without compression or friction upon the foot. They are as good shape after being worn six months as after having worn them one day. L. Baily.

Brooklyn, Nov. 24th, 1874.

From a somewhat extended observation of deformed and tender feet, and of boots and shoes as they are ordinarily made, it is apparent that *deformed feet* (*i. e.*, a departure from the normal healthy foot) are very common, and that the coverings usually made fail to correct, and serve only to increase, tenderness and deformity.

What is needed are shoes fitted to the natural foot. This work I believe the McComber last is doing, and in that belief I recommend it as the only practical application of correct principles. Jerome Walker, M.D.

I desire to say that I am earnestly laboring to introduce the improvement described on page 466 of this number of Hall's Journal of Health, and that I am having the most gratifying success in my business. Upon these lasts I construct all kinds of boots and shoes, and guarantee comfort and satisfaction to all ages and to both sexes. The fact is established by my system, that elegance in apparel for the feet is entirely compatible with comfort.

Parties at a distance who desire to avail themselves of the advantages of my system, can address me by mail, and I will supply them with full instructions by which they can make perfect diagrams and measurements of their feet, so that a fit can be safely promised.

The price of lasts fitted to individual feet is $5.00 per pair. To manufacturers and boot and shoemakers, I would say, that I am ready to supply them with my lasts in any quantity and for all styles of work direct from my factories. I am rapidly educating the intelligent public to a comprehension of the advantages of my last, and am satisfied that boot and shoemakers will be compelled to meet the demands of an enlightened public. Circulars, terms and all particulars will be sent on application.—Joel McComber, 21½ Spruce, and 197 & 189 William Streets, New York.

